# A20/TNFAIP3 Discriminates Tumor Necrosis Factor (TNF)-Induced NF-κB from JNK Pathway Activation in Hepatocytes

**DOI:** 10.3389/fphys.2017.00610

**Published:** 2017-08-23

**Authors:** Federico Pinna, Michaela Bissinger, Katharina Beuke, Nicolas Huber, Thomas Longerich, Ursula Kummer, Peter Schirmacher, Sven Sahle, Kai Breuhahn

**Affiliations:** ^1^Molecular Hepatopathology, Institute of Pathology, University Hospital Heidelberg Heidelberg, Germany; ^2^Institute of Pathology, RWTH Aachen University Hospital Aachen, Germany; ^3^Department of Modeling of Biological Processes, Centre for Organismal Studies, BioQuant, University of Heidelberg Heidelberg, Germany

**Keywords:** hepatocyte, apoptosis, computational modeling, decision-making process, hepatocellular carcinoma

## Abstract

In the liver tumor necrosis factor (TNF)-induced signaling critically regulates the immune response of non-parenchymal cells as well as proliferation and apoptosis of hepatocytes *via* activation of the NF-κB and JNK pathways. Especially, the induction of negative feedback regulators, such as IκBα and A20 is responsible for the dynamic and time-restricted response of these important pathways. However, the precise mechanisms responsible for different TNF-induced phenotypes under physiological stimulation conditions are not completely understood so far. In addition, it is not known if varying TNF concentrations may differentially affect the desensitization properties of both pathways. By using computational modeling, we first showed that TNF-induced activation and downstream signaling is qualitatively comparable between primary mouse hepatocytes and immortalized hepatocellular carcinoma (HCC) cells. In order to define physiologically relevant TNF levels, which allow for an adjustable and dynamic NF-κB/JNK pathway response in parenchymal liver cells, a range of cytokine concentrations was defined that led to gradual pathway responses in HCC cells (1–5 ng/ml). Repeated stimulations with low (1 ng/ml), medium (2.5 ng/ml) and high (5 ng/ml) TNF amounts demonstrated that JNK signaling was still active at cytokine concentrations, which led to dampened NF-κB signaling illustrating differential pathway responsiveness depending on TNF input dynamics. SiRNA-mediated inhibition of the negative feedback regulator A20 (syn. TNFAIP3) or its overexpression did not significantly affect the NF-κB response. In contrast, A20 silencing increased the JNK response, while its overexpression dampened JNK phosphorylation. In addition, the A20 knockdown sensitized hepatocellular cells to TNF-induced cleavage and activity of the effector caspase-3. In conclusion, a mathematical model-based approach shows that the TNF-induced pathway responses are qualitatively comparable in primary and immortalized mouse hepatocytes. The cytokine amount defines the pathway responsiveness under repeated treatment conditions with NF-κB signaling being dampened ‘earlier’ than JNK. A20 appears to be the molecular switch discriminating between NF-κB and JNK signaling when stimulating with varying physiological cytokine concentrations.

## Introduction

The role of the cytokine *tumor necrosis factor* (TNF) in the liver has been investigated intensively. Biological functions of TNF and subsequent activation of the NF-κB signaling pathway are associated with inflammatory processes, hepatocyte proliferation in response to acute or chronic liver damage, as well as tumorigenesis (DiDonato et al., 2012).

The hepatocellular response upon TNF stimulation is based on a sequence of post-translational modifications occurring downstream of the *TNF receptor* (TNFRI) with several proteins organized in the signalosome (Ruland, 2011). Binding of TNF to TNFRI sequentially activates the *mitogen-activated protein kinase kinase kinase 7* (MAP3K7/MEKK7, syn: TAK1) followed by the recruitment of *receptor-interacting serine/threonine-protein kinase 1* (RIPK1) and additional scaffold proteins (e.g., TAB, TRAF) to the signalosome. Transiently activated TAK1 is then mediating the phosphorylation of both IKKß and MKK4/7 leading to the activation of NF-κB and p38/JNK signaling, respectively (Wullaert et al., 2006). Importantly, direct transcriptional NF-κB targets, such as TNF α*-induced protein 3* (TNFAIP3, synonym: A20) or *nuclear factor of kappa light polypeptide gene enhancer in B-cells inhibitor*, α (IκBα) are essential for the fast and efficient shut-down of this pathway in terms of a negative feedback regulation (Ruland, 2011).

IκBα and A20 interfere with the TNF-induced response at different levels. While IκBα binds and inactivates the NF-κB dimers containing the subunit p65, A20 terminates the pathway response upstream of the IKK complex and therefore affects both the NF-κB and JNK response (Ruland, 2011). Remarkably, A20 contains both a deubiquitinase domain catalyzing K63 ubiquitin cleavage from RIPK1 and an E3 ubiquitin ligase domain facilitating K48 ubiquitin binding, which is associated with RIPK1 degradation (Ma and Malynn, 2012). Depending on the cell type these feedback mechanisms efficiently desensitize the TNF-induced NF-κB axis for 60–100 min after stimulation with TNF (refractory range) (Ashall et al., 2009). Notably, recent data demonstrated that the refractory time is not static. Instead this phase represents an adjustable system, which is regulated in an A20-dependent manner with direct impact on the secretome of cells and the authors hypothesized that this dynamic system is part of an inherent mechanism controlling the cellular inflammatory response (Adamson et al., 2016). However, it is unclear if and how existing feedback mechanisms differentially affect the downstream effector pathways induced by TNF in order to induce adjustable NF-κB and JNK responses.

NF-κB signaling exerts anti-apoptotic and cyto-protective properties, which is illustrated by many genetic *in vivo* models, in which deficiency of central pathway constituents, such as TAK1, IKKß, and IKKγ induced massive hepatocellular cell death (Luedde and Schwabe, 2011). This apoptosis in the absence of NF-κB depends on the persistent activation of the JNK axis followed by the production of reactive oxygen species (ROS) (Chen et al., 2003). Interestingly, previous experiments demonstrated that TNF activated NF-κB and JNK signaling with different dynamics (Iqbal and Zaidi, 2008), suggesting the existence of intra-cellular decision making processes discriminating between cyto-protection *via* NF-κB and programmed cell death *via* JNK. However, the functional duality of this system is a matter of debate and precise mechanisms regulating hepatocellular cell fate are not well understood.

The interpretation of the TNF input by the receptor/signalosome complex is central to understand this type of decision-making process. For example, a previous study showed that *apoptosis signal-regulating kinase 1* (ASK1) is involved in the TNF-mediated induction of persistent JNK activity and induction of apoptosis (Tobiume et al., 2001). These data suggest that the type of cytokine input (single vs. repeated stimulation) may affect TNF-downstream effectors and subsequently cell biology. How the mode of pathway activation modulates the cellular behavior in terms of a decision making process has not been analyzed, yet.

In this study we want to answer how variable and multiple TNF stimulations may adjust the NF-κB and JNK pathway response in hepatocellular cells. For this, we first combine experimental data and mathematical modeling to compare the dynamic NF-κB and JNK pathway response in primary hepatocytes and liver cancer cells. Second, the differential NF-κB and JNK pathway responses upon single and multiple TNF activation were analyzed. Lastly, the impact of the negative feedback regulator A20 as molecular switch on pathway activity and cell functionality was determined.

## Materials and methods

### Cell culture and TNF time courses

All experiments were performed in accordance with the institutional regulations. Murine hepatoma cell lines Hepa1-6 and Hep56 (CLS, Eppelheim, Germany) were seeded at a density of 4.0 × 10^5^ cells per 6 cm^2^ dish (TPP, Trasadingen, Switzerland) and cultivated at 37°C in DMEM-medium +10% FCS and +1% penicillin/streptomycin (Sigma Aldrich, Taufkirchen, Germany) for 24 h. Three hours prior to TNF stimulation, medium was removed, cells were washed with DMEM-medium and further cultivated in medium without FCS. For single stimulation of cells, 10 ng/ml recombinant murine TNF (R&D Systems, Minneapolis, USA) was used. For triple stimulations, 1 ng, 2.5 ng, and 5 ng/ml (time points 0, 60, and 120 min) were added to the medium. Medium was removed after 5, 10, 20, 40, 60, 120, 180, 240, 300, 360, 420, and 480 min and cells were washed with 1 × PBS (Life Technologies, Darmstadt, Germany) before protein and mRNA lysates were isolated. The MTT viability assay was performed as previously described (Malz et al., 2014).

### RNA-interference and expression vector transfection

Hepa1-6 cells were seeded at 1.5 × 10^5^ on 6 cm^2^ dishes 24 h prior to transfection. RNA-interference experiments were performed using the cationic carrier Oligofectamin (Life Technologies) according to the manufacturer's protocol. Each experiment included gene specific siRNA for A20 (GGG UAG GUU UGA AGA CUU A-dTdT) and Scramble siRNA (UGG UUU ACA UGU CGA CUA A-dTdT, Thermo Fisher Scientific, Ulm, Germany; final concentration: 100 nM). Fourty-eight hours after siRNA-transfection, cells were used for TNF time course experiments.

For vector transfection, Hepa1-6 cells were seeded at 1.5 × 10^5^ on 6 cm^2^ dishes 24 h before transfection. Attractene was used according the manufacturers' protocol (Qiagen, Hilden, Germany). For transfection experiments, A20 was cloned into the pCMV6-Entry vector creating a A20-myc-ddk fusion transcript (mTNM_001166402; Origene, Frankfurt, Germany). Fourty-eight hours after vector transfection, cells were used for TNF time course experiments. The pCMV6-A20 vector was validated by sequencing. For transfection efficiency estimation, cells were transfected with pMaxvector according to the manufacturer's instructions (Lonza, Walkersville, USA). Forty-eight hours after transfection GFP-positive cells were determined by FACS analysis.

### Preparation of total mRNA and real-time PCR

Total mRNA was isolated using the NucleoSpin RNA kit according to the manufacturers‘ protocol (Macherey-Nagel, Dühren, Germany). One microgram total mRNA was used for cDNA-synthesis using RevertAid H Minus Reverse Transcriptase and random primers (LifeTechnologies). For semiquantitative evaluation of mRNA the real-time PCR ABsolute qPCR SYBR Green ROX Mix was used (Thermo Fisher Scientific). The following cycling program was applied: 95°C denaturation for 15 min followed by 40 cycles 95°C/15 s and 60°C/1 min. Successful PCR reaction was tested by a melting curve analysis: 95°C/15 s, 60°C/30 s, and 95°C/15 s. The following primers were used in our study: mA20-forward: 5′-TTC CAC TTG TTA ACA GAG AC-3′, mA20-reverse: 5′-TAC TCC TTT AGA AGC TTT TC-3′, mIκBa forward: 5′-CCT GGC CAT CGT GGA GCA CT-3′, mp65-forward: 5′CCG GAC TCC TCC GTA CGC CG-3′, mp65- reverse: 5′CTT GAA GGT CTC ATA GGT CC-3′, mIκBa reverse: 5′-AGT AGC CTT GGT AGG TTA CC-3′, mtubulin forward: 5′-TCA CTG TGC CTG AAC TTA CC-3′; mtubulin reverse: 5′-GGA ACA TAG CCG TAA ACT GC-3′ (Thermo Fisher Scientific).

### Protein isolation and western immunoblotting

Proteins were collected after TNF stimulation using the Cell Lysis buffer (Cell Signaling Technology, Frankfurt, Germany) supplemented with PhosStop (Roche, Mannheim, Germany) as well as Protease Inhibitor Cocktail Mix G (Serva, Heidelberg, Germany) and stored in liquid nitrogen. After thawing, samples were sonicated (3 times for 30 s) and pelleted by centrifugation (10 min, 16,100 g at 4°C). Protein amounts were measured with the Nanodrop spectrophotometer (Thermo Scientific). One hundred-fifty microgram of total protein per lane were loaded on a 8% PAA/SDS gel. Proteins were blotted on Nitrocellulose membrane (Protran B, GE Healthcare Lifesciences, Freiburg, Germany) and blots were incubated in a 5% milk powder/TBS-T solution containing the respective primary antibody overnight at 4°C. After washing with TBS-T, membranes were incubated with the secondary antibody (5% milk powder/TBS-T) at room temperature for 1 h.

The following antibodies were used in this study. Primary antibodies: anti-actin (dilution: 1:10,000, MP Biomedical, Eschwege, Germany), anti-A20 (dilution: 1:200, Santa Cruz Biotechnology, Heidelberg, Germany), anti-caspase-3 (1:500, Cell Signaling Technology), anti-IκBα (dilution: 1:500, Cell Signaling Technology), anti-phospho-IκBα (Ser32, dilution: 1:500, Cell Signaling Technology), anti-SAPK/JNK (1:500, Cell Signaling Technology), anti-phospho-SAPK/JNK (1:500, Cell Signaling Technology), anti-p65 (dilution 1:200, Santa Cruz Biotechnology), anti-phospho-p65 (Ser536, 1:500, Cell Signaling Technology), TNFR1 (dilution 1:200, clone: H-271, Santa Cruz), ASK1 (clone: D11C9, Cell Signaling Technology).

Secondary antibodies: donkey anti-rabbit (IRDye coupled, 800 CW, dilution: 1:1,000), donkey anti-mouse (IRDye coupled, 800 CW, dilution: 1:1,000) and donkey anti-mouse (IRDye coupled, 680 LT, dilution: 1:20 000, all antibodies from LI-COR Biosciences).

### Co-immunoprecipitation

Cells (Hepa1-6) were grown to 80% confluence and stimulated with TNF (10 ng/ml) for 10, 20, 60, and 120 min. Proteins were isolated using NP40 buffer (50 mM Tris-HCl, 150 mM NaCl, 1% NP40). Total cell lysate was pre-cleared with 15 μl/sample GammaBind™ G Sepharose™ (GE Healthcare, Germany). Five milligrams of total proteins were incubated with 6 μg of primary antibody recognizing ASK1 for 2 h under continuous rotation at 4°C. Thirty-five microliters of beads (Protein A-Agarose, Santa Cruz Biotechnology) were added to each sample and incubated overnight. Beads were washed with NP40 buffer two times, diluted in sample buffer, and used for Western immunoblotting.

### Luciferase reporter gene assay

Luciferase gene reporter assays were performed as previously described (Weiler et al., 2017). Cells were transfected with plasmids containing NF-κB promoter elements fused to Firefly luciferase (pNF-κB-luc, Agilent, Waldbronn, Germany) and with Renilla Luciferase (pRL-CMV, Promega, Mannheim, Germany) at a ratio of 2:1 using Fugene HD transfection reagent (Promega) according to the manufacturer's instructions. Luciferase activity was measured 48 h after transfection using the Dual-Luciferase Reporter Assay System (Promega). Firefly luciferase activity was normalized to Renilla Luciferase activity. As positive control a plasmid containing NF-κB-activator MEKK was used (pFC-MEKK, Agilent, Waldbronn, Germany).

### Caspase-3 and apoptosis assay

Protein lysates were harvested using the Caspase Lysis buffer (20 mM Tris pH 7.4, 137 mM NaCl, 2 mM EDTA, 10% glycerol, 1% Triton X-100), pelleted (10 min, 16,100 *g*, 4°C) and protein concentration was quantified using Bradford Assay. Fifty microgram of protein extracts were incubated with a tetrapeptide fluorogenic substrate specific for Caspase-3 (50 μM Ac-DEVD-AFC, Enzo Life Science, Lausen, Switzerland) diluted in Caspase Assay buffer (50 mM HEPES, 50 mM NaCl, 10 mM EDTA 10 mM 1,4-dithio-DL-threitol, 0,1% CHAPS buffer, 5% glycerol) for 1 h. Caspase-3 activity was immediately measured using a fluorescent microplate reader (excitation 405 nm/emission 530 nm, FLUOstar Omega, BMG Labtech, Ortenberg, Germany).

For the measurement of early apoptosis, the Guava Nexin Reagent was used according to the manufacturer's protocol 24 h after transfection (Millipore/Merck KGaA, Darmstadt, Germany). As positive controls cells treated with 1 μM Doxorubicin for 24 h were used. The Guava easyCyte HT was used for all measurements (Millipore/Merck KGaA).

### Data analysis and statistics

Western immunoblotting and qPCR experiments were performed in 2–3 technical replicates. For each data point derived from Western immunoblotting, the relative protein amounts were quantitatively measured using the Image Studio software (LI-COR Biosciences). Values obtained from each measured protein sample were normalized to the respective actin value. Data are presented as mean ± standard errors. Statistical comparisons between two groups were done using the non-parametric *t*-test (IBM, SPSS software, Armonk, NY, USA).

### Mathematical pathway modeling

In order to substantiate our assumption of equivalence between primary hepatocytes and immortalized hepatocellular carcinoma (HCC) cells we decided to perform a quantitative modeling approach to test whether an established model for primary hepatocytes can be adapted to fit the data from Hepa1-6 and Hep56 cells (Beuke et al., 2017). The methodological assumption was that the model represented and integrated quantitative information from previous experiments. The ODE-based model was originally created to describe data from primary mouse hepatocytes after TNF stimulation (Pinna et al., 2012) and was extended and tested to qualitatively describe time-resolved, dynamic response of NF-κB signaling to various doses of TNF (Beuke et al., 2017). To confirm equivalence of processes in the different cell types it was required that the mathematical model could be adjusted to the observations in Hepa1-6 cells without changes in the model structure or to parameters describing biochemical properties of the molecules, such as Km values. Justifiable model adjustments were changes in expression levels or turnover rates of signaling compounds or changes in transcription or translation rates. Since the original model comprehensively described the known relevant processes in the canonical NF-κB pathway it was relatively large (28 variables, 49 reactions) and consequently its parameter values were not completely identifiable. Therefore, we worked with ensembles of model parameterizations that each describe the experimental data well and that allow the generation of predictions even without complete parameter identifiabilty (Beuke et al., 2017). The model simulations and parameter estimations were carried out in COPASI (Mendes et al., 2009).

## Results

### Hepatocellular response of NF-κB and JNK pathway activation upon TNF stimulation

We recently developed a computational *ordinary differential equation* (ODE) model for TNF-induced NF-κB activation based on quantitative and time-resolved experimental data derived from primary murine hepatocytes (Pinna et al., 2012). However, functional analysis and genetic manipulation of primary hepatocytes are technically challenging; primary hepatocytes are difficult to transfect and even minor contamination with other liver cell types (e.g., Kupffer cells) may compromise data quality and interpretation. To test if immortalized murine HCC cells (Hepa1-6) can be used as functional model for non-malignantly transformed hepatocytes, we utilized a computational approach to compare the TNF-induced NF-κB and JNK pathway activation.

**Figure 1:**
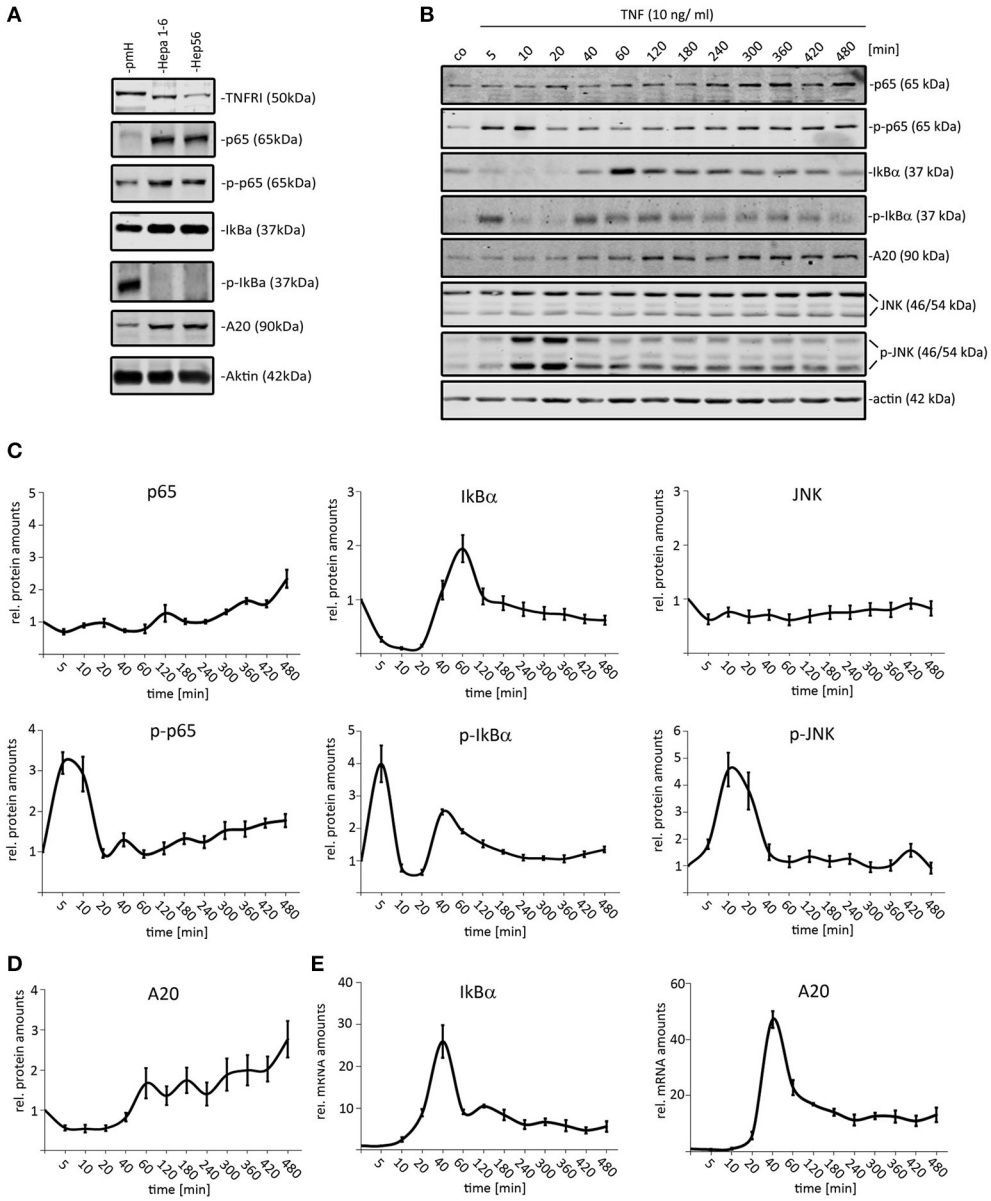
Dynamic activation of TNF-induced NF-κB and JNK pathways in mouse liver cancer cells. **(A)** Comparison of p65/phospho-p65, IκB-α/phospho-IκB-α, TNFR1, and A20 protein amounts in primary murine hepatocytes (pmH) and 2 mouse HCC cell lines (Hepa1-6, Hep56). The mean TNFR1 difference between both HCC cell lines and hepatocytes was calculated based on three independent experiments followed by signal quantification. **(B)** Exemplary Western immunoblots of protein extract isolated from Hepa1-6 cells after administration of TNF (10 ng/ml) for the indicated time-points (untreated and 5–480 min after stimulation). Signals for p65/phospho-p65, IκBα/phospho-IκBα, JNK/phospho-JNK, and the negative feedback regulator A20 were quantified and normalized to the respective loading control (actin). **(C)** Relative protein amounts of NF-κB and JNK pathways constituents in Hepa1-6 cells after single TNF administration (10 ng/ml). **(D)** Relative protein amounts of A20 in Hepa1-6 cells after single TNF administration (10 ng/ml). **(E)** Relative mRNA levels of A20 and IκBα after single TNF stimulation. Graphs in **(C–E)** summarize the results from three independent experiments. Bars in panel **(C–E)** represent standard errors.

Analyzing the expression of selected NF-κB pathway constituents revealed that both tested HCC (Hepa1-6 and Hep56) cell lines expressed higher p65 and IκBα protein amounts than primary hepatocytes (Figure 1A). In contrast, the total concentration of the TNFR1 was reduced in both HCC cells compared to non-malignantly transformed cells (median change: 2.5x). We then stimulated Hepa1-6 cells with TNF (10 ng/ml) and the total protein extracts were analyzed by quantitative Western immunoblotting for up to 480 min (12 time points; Figures 1B–D). As read-out, the activation of TNF downstream effectors, such as NF-κB (total p65, phospho-p65) and JNK (total JNK/phospho-JNK) was analyzed. In addition, the expression of the known negative feedback regulators IκBα (mRNA, protein, phosphorylation) and A20 (mRNA and total protein) was evaluated. For p65 and JNK an activation/phosphorylation with peaks around 5–10 min was detected in Hepa1-6 cells (Figures 1B,C). Total levels of IκBα showed an immediate decrease due to fast phosphorylation and concomitant proteasomal degradation followed by quick recovery to initial and higher concentrations (Figures 1B,C). A20 protein levels steadily increased 40–60 min after TNF stimulation until the end of the experiment (Figures 1C,D). In addition, real-time PCR results illustrated that the transcription of both feedback regulators was induced around 40 min after TNF administration, while the mRNA expression of other pathway constituents, such as p65 and IKKs was not affected (Figure 1E and data not shown). A similar dynamic pathway response was detected for another HCC cell line (Hep56, Supplementary Figure S1).

**Figure 2:**
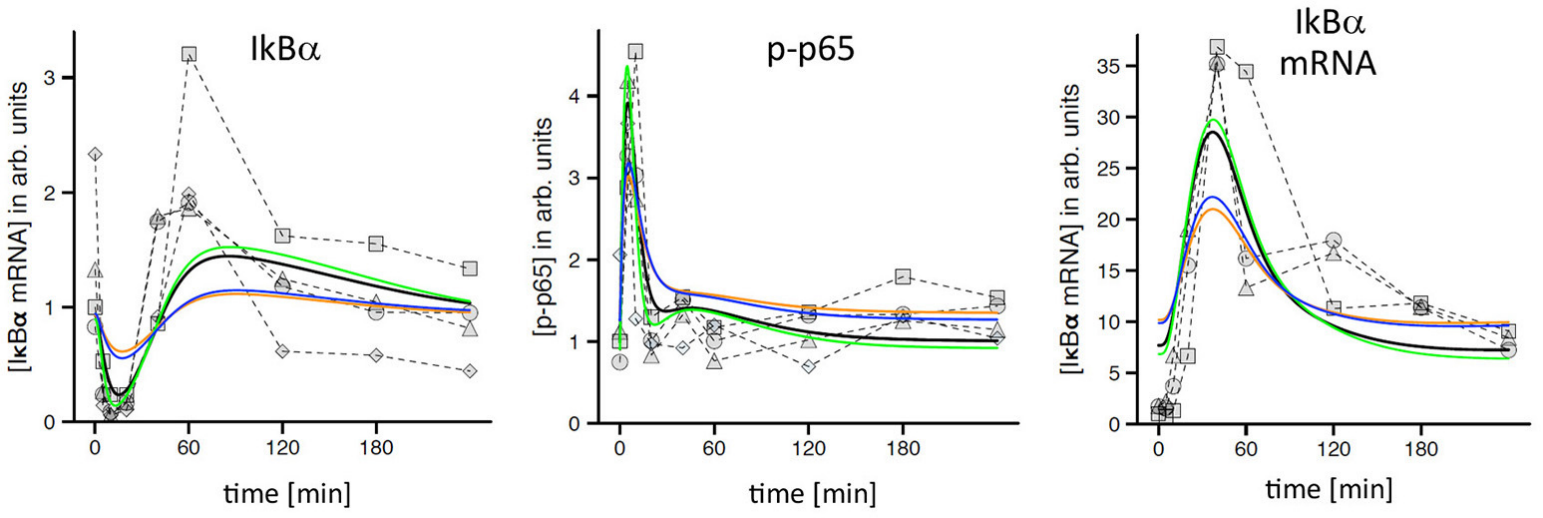
TNF-induced NF-κB response in Hepa1-6 cells and model simulations. Measured time courses of IκBα, phospho-p65, and IκBα-mRNA after administration of 10 ng/ml TNF (symbols connected by dashed lines indicate replicates from independent experiments). Corresponding simulations (black lines) were obtained from a model that was originally created and calibrated for murine primary hepatocytes and only slightly readjusted to fit the Hepa1-6 data (Beuke et al., 2017). Yellow lines: simulations after reduction of TNFR1 by a factor of 2.5. Blue lines: simulations after adjustment of IKK expression. Green lines: simulations after adjustment of TNF/TNFR1 internalization.

A first visual comparison of data derived from primary hepatocyte data and Hepa1-6 cells already illustrated a high degree of qualitative similarities between both cell types (Figure 1; Pinna et al., 2012). In addition, previously established mathematical models derived from primary hepatocytes were used for quantitative testing of HCC cell line kinetics (Pinna et al., 2012; Beuke et al., 2017) Model predictions were compared to measurements of IκBα, phospho-IκBα, phospho-p65, and IκBα mRNA after TNF stimulation in Hepa1-6 cells. Adjusting the IκBα transcription rate was necessary to obtain satisfying model fits with comparable quality as observed for primary cells (Beuke et al., 2017; Figure 2, black lines).

Interestingly, simulations after the reduction of total TNFR1 levels as indicated by our initial comparison of HCC cells and hepatocytes (Figure 1A, factor: 2.5) revealed a clear pathway dampening with regard to IκBα, phospho-p65, and IκBα mRNA amplitudes (Figure 2, yellow lines). Because no obvious differences between tumor cells and primary cells were detected in our experimental data sets, we hypothesized that adjustments of other pathway constituents can compensate the attenuated responsiveness upon TNFR1 reduction. The previously satisfying fit could be restored by applying simple parameter changes in the model either for the receptor dynamics (e.g., the internalization rate of the activated receptor complex; Figure 2, green lines) or at the level of IKK (e.g., IKK expression levels and IKK activation rate constants, data not shown). Other adjustments led to partial rescue of the dynamic pathway response (e.g., exclusive adjustment of IKK expression levels; Figure 2, blue lines). These results strongly suggested that hepatocellular (tumor) cells potentially harbor a number of adjustable setscrews, which may compensate for receptor variations. In addition, we drew the conclusion that most involved processes were qualitatively similar between hepatocytes and HCC cells.

In sum, the dynamic activation of all analyzed signaling pathway and feedback constituents in HCC cells corresponded qualitatively between primary hepatocytes and immortalized HCC cells. Therefore, HCC cells represent a suitable *in vitro* model for the study of TNF-induced activation of NF-κB and JNK signaling as well as for the dynamic pathway activation after genetic manipulation.

### Defining the responsive range of hepatocellular cells to TNF

In most studies analyzing TNF-induced signaling, cells were stimulated with high cytokine concentrations (10–50 ng/ml) (Werner et al., 2008; Ashall et al., 2009; Turner et al., 2010; Wang et al., 2011). In the liver these TNF concentrations may only be detectable under specific patho-physiological conditions, such as non-alcoholic steatohepatitis (Krawczyk et al., 2009). Depending on the disease and used detection method, TNF amounts around 30 ng/ml or even higher concentrations could be measured (Krawczyk et al., 2009). However, it is unknown if lower TNF concentrations are able to induce an adjustable cellular response in hepatocytes.

**Figure 3:**
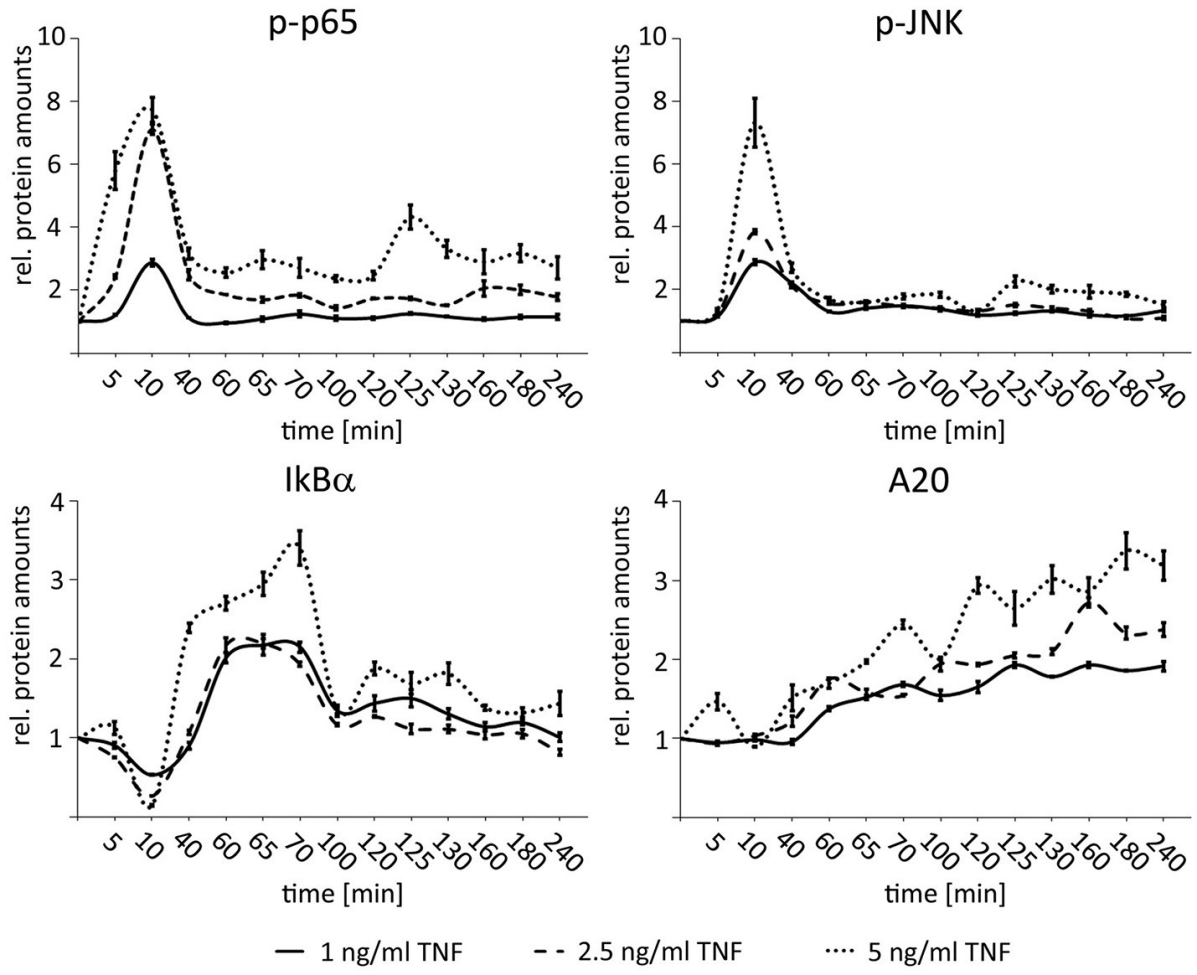
Dose-dependent activation of NF-κB and JNK signaling after TNF stimulation in HCC cells. Graphs summarizing the results of Western immunoblots analysis after treatment of Hepa1-6 cells with TNF (1, 2.5, and 5 ng/ml) for the indicated time-points (untreated and 5–240 min after stimulation). Signals for phospho-p65, phospho-JNK, IκBα, and A20 were quantified and normalized to the respective loading control (actin). Graphs summarize the results from three independent analyses. Bars represent standard errors.

In order to define the dynamic range of NF-κB and JNK responsiveness in HCC cells with regard to different TNF concentrations, dose response experiments were performed with low-level but physiologically relevant TNF concentrations. For this Hepa1-6 cells were treated with 1, 2.5, and 5 ng/ml TNF and analyzed for the expression of p65 (total p65 and phopsho-p65), IκBα (total IκBα and phospho-IκBα), and JNK (total JNK and phospho-JNK) for up to 240 min. All cytokine concentrations induced specific phospho-p65 and phospho-JNK pathway responses, however, with concentration-dependent amplitudes (Figure 3). Even lowest TNF concentrations led to a sufficient expression of both negative feedback regulators IκBα and A20. Higher TNF concentrations (>5 ng/ml) did not further increase the NF-κB or JNK responses indicating pathway saturation (Beuke et al., 2017).

These results illustrate that 1–5 ng/ml TNF cover a cytokine range in which the hepatocellular cells can respond differentially to varying input information.

### TNF-induced negative feedback differentially blocks NF-κB and JNK signaling

**Figure 4:**
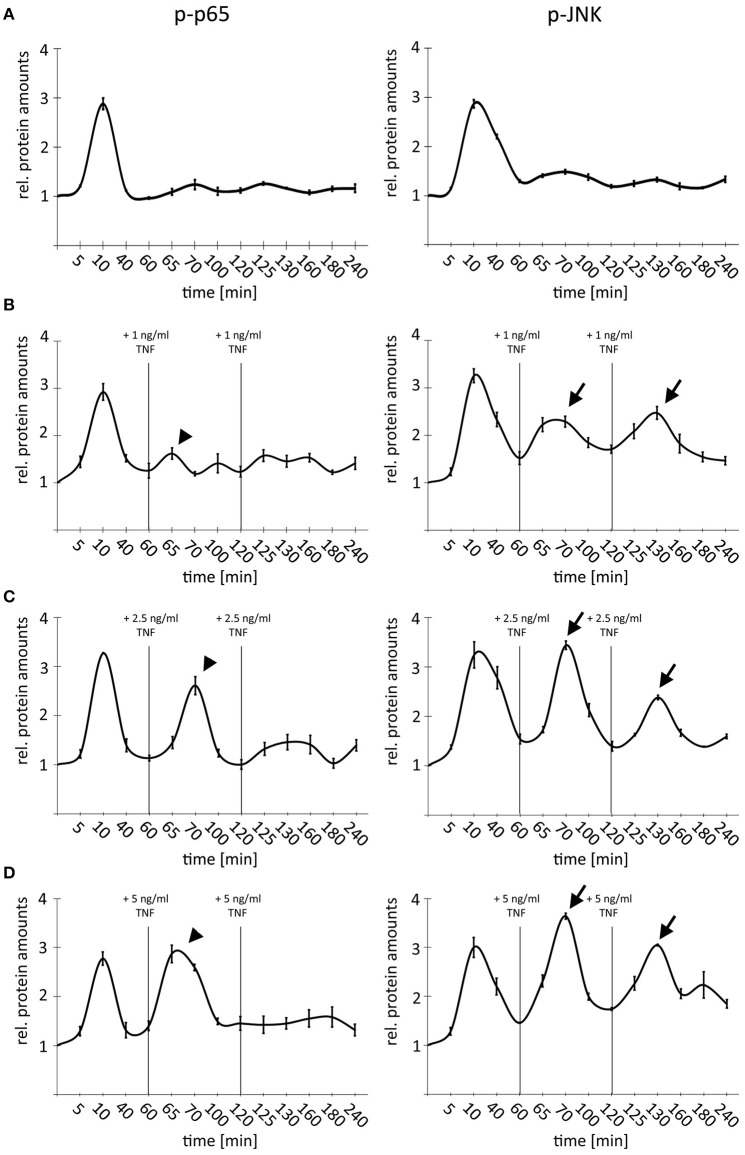
Comparison of NF-κB and JNK pathway activity after single and multiple TNF stimulation. **(A)** Single TNF treatment of Hepa1-6 cells (1 × 1 ng/ml). **(B)** Triple TNF treatment Hepa1-6 cells with 3 × 1 ng/ml. **(C)** Triple TNF treatment Hepa1-6 cells with 1 × 1 ng/ml followed by 2 × 2.5 ng/ml. **(D)** Triple TNF treatment Hepa1-6 cells with 1 × 1 ng/ml followed by 2 × 5 ng/ml. For all graphs signals for p65/phospho-p65, and JNK/phospho-JNK were quantified and normalized to the respective loading control (actin). For **(B–D)** additional treatments and respective TNF concentrations are indicated. Arrowheads indicate p-p65 responses while arrows indicate p-JNK responses. Graphs summarize the results from three independent analyses. Bars represent standard errors.

Treatment of very high TNF amounts (10–50 ng/ml) led to an efficient desensitization of cells due to activation of negative feedback regulators (Ashall et al., 2009). In order to analyze the impact of TNF concentrations in the dynamic range between 1 and 5 ng/ml on this desensitization, a time-course experiment was designed, by which we combined multiple-pulse treatments with different doses of TNF (1 × 1 ng/ml; 3 × 1 ng/ml, 1 × 1 ng/ml followed by 2 × 2.5 ng/ml; 1 × 1 ng/ml followed by 2 × 5 ng/ml) (Figure 4). Cells were repeatedly stimulated with TNF after 60 and 120 min based on published data illustrating a restoration of pathway sensitivity after this time period (Ashall et al., 2009). As expected, a single TNF pulse induced a temporary activation of NF-κB and JNK signaling with comparable amplitude (Figure 4A). This first stimulation was sufficient to completely block p65 but not JNK phosphorylation after adding additional low doses of TNF (2 × 1 ng/ml; Figure 4B, arrows and arrowheads). Higher TNF concentrations (2 × 2.5 ng/ml) were able to partly overcome the refractory behavior and efficiently stimulated the phosphorylation of p65; however, the JNK response was always stronger after the second and third cytokine administration (Figure 4C, arrows and arrowheads). Lastly, repeated treatment with highest additional TNF concentrations (2 × 5 ng/ml) did not block the second phosphorylation of p65 but drastically dampened the third peak; however, a clear third induction of JNK was still detectable (Figure 4D, arrows and arrowheads). Importantly, identical results were obtained using another HCC cell line (Hep56, Supplementary Figure S2).

These data illustrate that under conditions of non-saturated stimulation, TNF differentially affected NF-κB and JNK signaling in hepatocellular cells. TNF-induced temporal desensitization supported JNK signaling when NF-κB signaling was still efficiently dampened.

### A20 discriminates between NF-κB and JNK pathway activation

A20 is a known inhibitor of NF-κB and JNK signaling; however, its point of interference differs from IκBα since it blocks TNF-responses at the signalosome level (Lee et al., 2000; Ruland, 2011). We therefore hypothesized that the NF-κB-induced expression of A20 (Figure 1D) could act as a rheostat for the differential inhibition of NF-κB and JNK signaling in hepatocellular cells.

**Figure 5:**
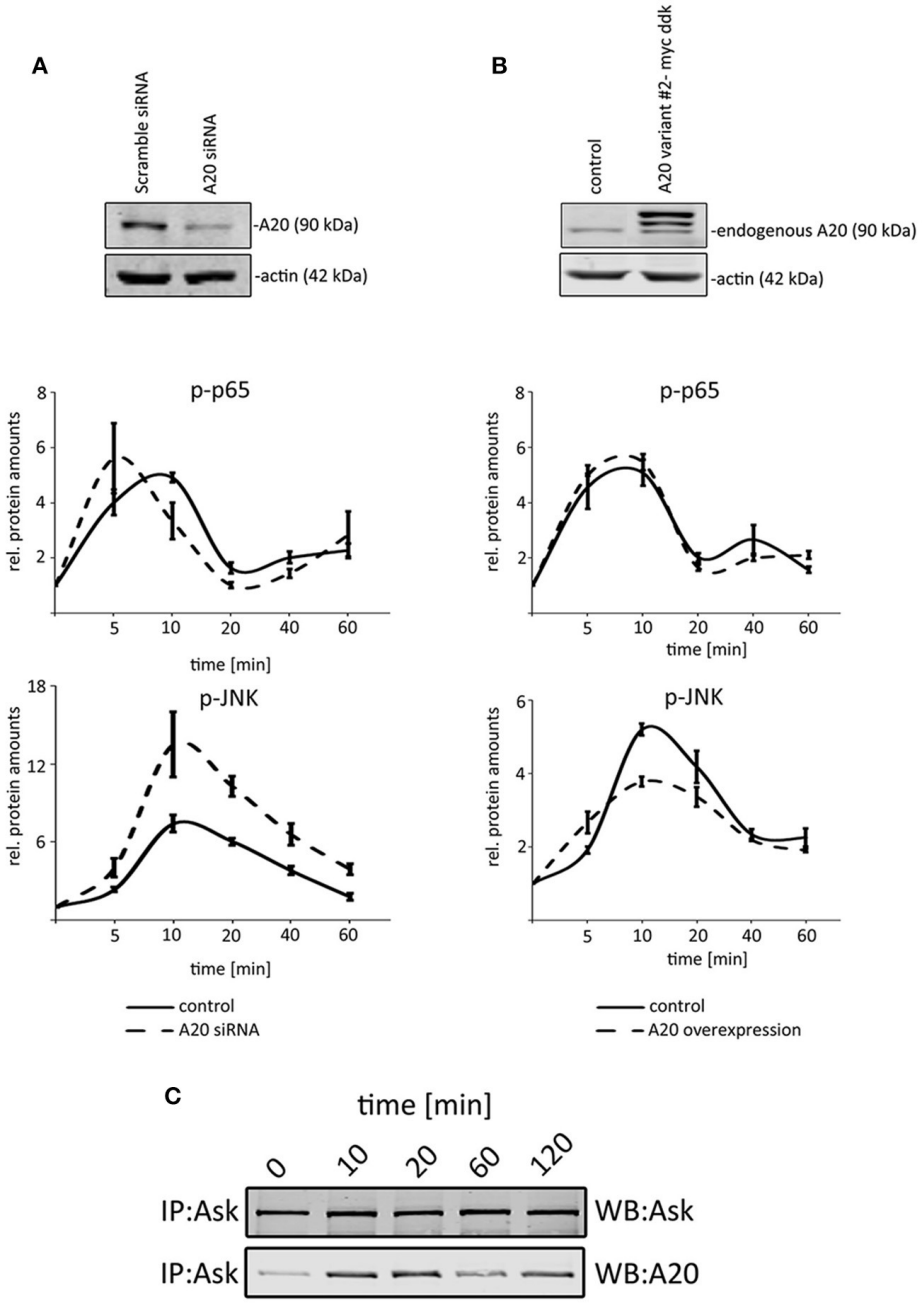
A20 negatively regulated JNK signaling in hepatocellular cells. **(A)** The overexpression of A20 is confirmed by western immunoblotting. Endogenous A20 and exogenous A20 were detected. Kinetics illustrate that NF-κB signaling is not affected after increasing A20 levels, while the phosphorylation of JNK is reduced after TNF administration (10 ng/ml). **(B)** The siRNA-mediated knockdown of A20 is confirmed by western immunoblotting. Kinetics after TNF administration (10 ng/ml) showed that JNK phosphorylation increased compared to controls. p65/phospho-p65 and JNK/phospho-JNK were quantified and normalized to the respective loading control (actin). All graphs summarize the results from three independent analyses. Bars represent standard errors. **(C)** Co-immunoprecipitation experiment detecting the physical interaction between A20 and ASK1 after TNF stimulation at indicated time points. The detection of ASK1 illustrates that the protein is not differentially expressed after TNF administration. IP: immunoprecipitation; WB: Western immunoblotting.

Because our previous data illustrated that TNF induced A20 protein expression after 40–60 min (Figure 1C), we decided to analyze the first 60 min after TNF administration to define the effects of basal A20 levels (without p65-induced A20 levels after 60 min). To test the impact of A20, we compared the activation of p65 and JNK after genetically changing the basal A20 levels in hepatocellular cells. Because the stable overexpression of A20 did not result in viable clones (Supplementary Figure S3A), we optimized the transfection protocol to achieve high transient transfection efficiencies of at least 80% (Supplementary Figures S3B,C). First, we transiently overexpressed murine A20 in Hepa1-6 cells and performed TNF stimulation experiments for up to 60 min (10 ng/ml; Figure 5A). While no significant effects on the phosphorylation of p65 were detectable, a reproducible reduction of JNK activation was observed (Figure 5B). *Vice versa*, the siRNA-mediated, specific inhibition of A20 again did not significantly affect the activation of p65. In contrast, JNK phosphorylation significantly increased in cells with transient silencing of A20. The fact that A20 did not affect p65 activity was confirmed in independent experiments including target gene expression and luciferase reporter assays (Supplementary Figures S4A,B). In order to further characterize the possible molecular basis for the differential impact of A20 on NF-κB and JNK, interaction studies were performed. Since ASK1 may sustain JNK activity and because A20 can interact with ASK1, we initiated co-immunoprecipitation experiments after TNF treatment (Tobiume et al., 2001; Won et al., 2010). Western immunoblotting revealed peculiar oscillatory dynamics between ASK1 and A20 with a maximum peak between 10 and 20 min after TNF stimulation (Figure 5C). Together with the published results, our data strongly suggested that the interaction between A20 and ASK1 is dynamically regulated by TNF and therefore might represent one possible mechanism how A20 controls JNK activity in the cell types analyzed here.

These data suggest that basal A20 concentrations differentially inhibited NF-κB and JNK signaling and therefore are likely to be involved in a modulation of the JNK response in phases of TNF-induced desensitization.

### A20 protects from a TNF-induced caspase3-cleavage

A20 is overexpressed in human HCCs illustrating that elevated A20 level may support tumorigenic properties of HCC cells (Chen et al., 2015; Catrysse et al., 2016; Wang et al., 2016). Because basal A20 abundance negatively regulated JNK pro-apoptotic phosphorylation (Figure 5), we hypothesized that decreased A20 levels can support hepatocellular programmed cell death.

**Figure 6:**
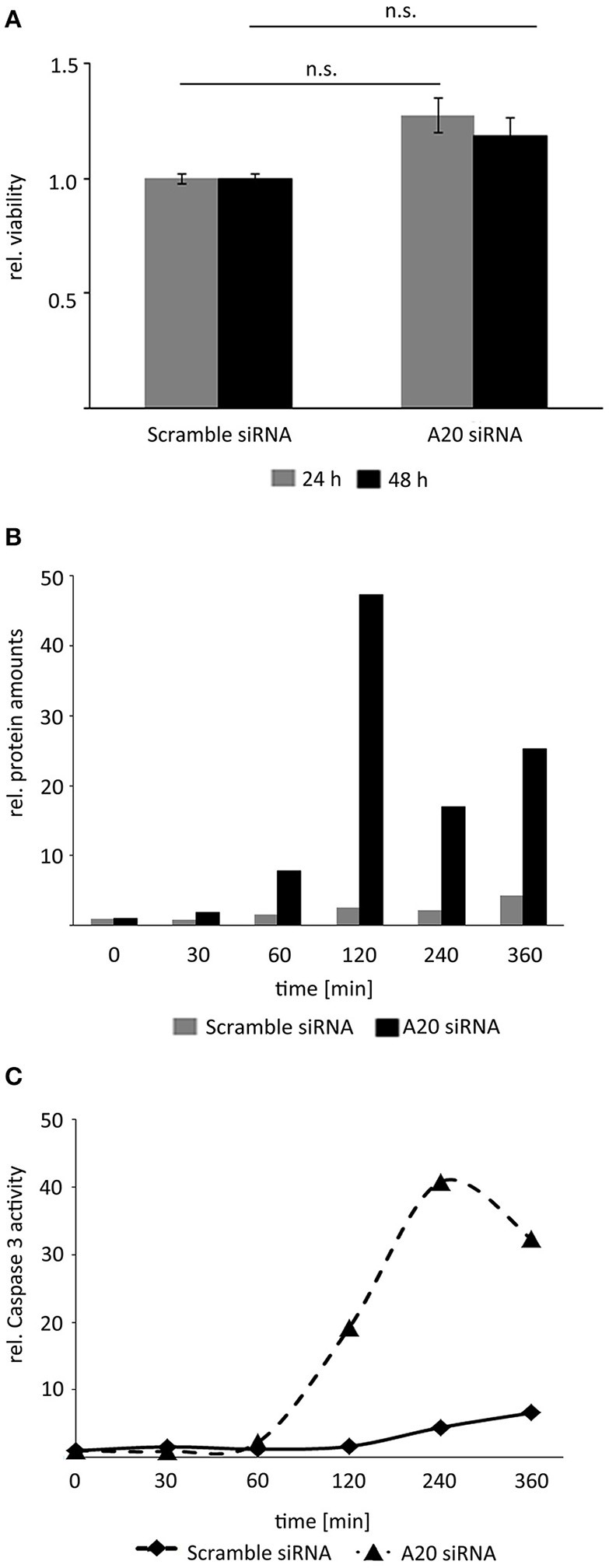
Reduction of A20 sensitizes hepatocellular cells to TNF-induced apoptosis. **(A)** MTT viability assay illustrated that inhibition of A20 by siRNA did not affect the viability of Hepa1-6 cells after 24 and 48 h. **(B)** Measurement of caspase-3 fragments after A20 inhibition and TNF stimulation by Western immunoblotting. Exemplary results after densitometric quantification are shown. An independent repetition led to similar results. **(C)** Measurement of caspase-3 activity after A20 silencing followed by TNF stimulation. Exemplary results after fluorometric measurement are shown. An independent repetition led to similar results.

Inhibition of A20 by siRNA alone did not affect HCC cell viability indicating that TNF stimulation and subsequent activation of the downstream pathways was necessary to uncover the biological effects of A20 (Figure 6A). In contrast, inhibition of A20 for 48 h and subsequent treatment with TNF induced cleavage of the effector caspase-3 around 120 min after cytokine administration as detected by Western immunoblotting (Figure 6B). Notably, this perturbation approach diminished both basal A20 levels and TNF-induced A20 levels (starting after 40–60 min). To confirm the negative effects of A20 on caspase-3, additional activity assays were performed after A20 perturbation for 48 h followed by TNF stimulation. As already indicated by caspase-3 cleavage experiments, caspase activity was significantly increased after reduction of A20 expression about 60 min after TNF treatment (Figure 6C). In contrast, no significant induction of apoptosis was detectable by FACS using an identical protocol (Supplementary Figure S5). This led us to the conclusion that the loss of A20 followed by TNF stimulation (and stabilization of the JNK pathway) is not sufficient to induce a full-blown apoptotic response, however, first steps toward apoptosis, such as activation of an effector caspase-3 may represent the molecular requirement for this process. These underlying mechanisms are currently under further investigation.

In summary, these results show that elevated A20 amounts reduced the activity of central effector caspases in hepatocellular cells after TNF stimulation.

## Discussion

The liver represents a frontline organ critically involved in the regulation of metabolic processes, hormone production, detoxification, and immunological responses. For this, a precise and temporary paracrine cross talk between non-parenchymal (Kupffer cells, liver sinusoidal endothelial cells, and hepatic stellate cells) and parenchymal liver cells (hepatocytes) is of central importance to fine tune and adjust the cellular and biological responses in these cell types. In this context, TNF-induced signaling is a key constituent of the innate immune response. For this, TNF is immediately produced by non-parenchymal cells in the liver e.g., in response to circulating pathogens, such as bacterial toxins (Seki et al., 2001; Wu et al., 2010). Next to its immune-modulatory properties, TNF regulates proliferation and apoptosis in hepatocytes. However, it is unclear how varying individual and continuous TNF administration affect molecular decision-making process and hepatocyte biology.

Induction of the NF-κB and/or JNK pathways by TNF has been intensively analyzed in different cancer cell types and immortalized fibroblasts under various culture conditions. In addition, computational modeling focused on the subcellular localization of NF-κB (Turner et al., 2010), the role of feedback mechanisms relevant for pathway termination (Werner et al., 2008), and how cytokine concentrations affect the oscillatory pathway behavior (Wang et al., 2011). Recently, computational modeling demonstrated that TNF, which is secreted by Kupffer cells and liver sinusoidal endothelial cells (LSECs) in response to lipopolysaccharide (LPS), induced an adjustable molecular response in primary isolated hepatocytes (Beuke et al., 2017). This mathematical model was robust for many model parameters indicating that changes of pathway constituents, such as IκBα did not significantly change the NF-κB pathway properties. This is supported by data presented in our study illustrating that this model with only slight modifications sufficiently explain the dynamic NF-κB behavior in primary hepatocytes and HCC cells, although initial amounts of p65 and IκBα are different (Figure 1A). To our knowledge, this is the first study demonstrating that TNF-induced NF-κB pathway dynamics is qualitatively comparable in normal cells and malignantly transformed cells.

Our results demonstrate that TNF in a range between 1 and 5 ng/ml differentially desensitize NF-κB and JNK signaling in hepatocyte-derived cells. We hypothesized that A20 represents a molecular switch discriminating between the two pathways in a physiological concentration range. Our experimental data revealed that A20 negatively regulates pro-apoptotic JNK signaling and therefore favors the pro-survival NF-κB axis. Interestingly, increased A20 levels are detectable in human HCC tissue compared to surrounding livers, and recent publications confirm the oncogenic properties of elevated A20 levels (Chen et al., 2015; Catrysse et al., 2016; Wang et al., 2016). In addition, A20 inhibition followed by TNF stimulation supports caspase-3 cleavage and its enzymatic activity suggesting a phenotype prone to apoptosis. These results were confirmed by previous publications showing that increased A20 protects hepatocytes from TRAIL-induced apoptosis or supports hepatocyte proliferation after partial hepatectomy (Longo et al., 2005; Dong et al., 2012).

Importantly, in our experimental setup we used TNF concentrations that can be detected in different areas of the liver vasculature network (3–10 ng/ml) under physiological and pathological conditions (Porowski et al., 2015). However, under some conditions, such as non-alcoholic steatohepatitis (NASH) or after liver transplantation much higher TNF levels may be measurable (34.2 and 43 ng/ml, respectively). This suggests that under pathological conditions differential desensitization of NF-κB and JNK signaling upon TNF stimulation is not effective anymore (Krawczyk et al., 2009; Fernandez-Yunquera et al., 2014), since high TNF concentrations abolished any fine-tune regulation of both pathways (Figure 4). The biological relevance of precise cytokine concentrations has been confirmed in studies showing that different TNF amounts can cause distinct responses with regard to target gene expression (Ashall et al., 2009). Moreover, our recent computational multi-scale model illustrated that LPS-induced TNF levels between 0.1 and 5 ng/ml cause an adjustable NF-κB pathway response, while higher cytokine amounts show maximal pathway amplitudes (Beuke et al., 2017).

In addition, our results might be of clinical relevance for patients with continuous inflammatory responses (e.g., during hepatitis). The data suggest that repeated induction of low TNF amounts (between 1 and 2.5 ng/ml) can already lead to stronger pro-apoptotic JNK activation but less-pronounced pro-survival NF-κB signaling. Thus, it is tempting to speculate that inhibition of the JNK pathway in earliest phases of liver damage might prevent recurrent cycles of cell death followed by regenerative proliferation, which is one characteristic of e.g., chronic hepatitis C virus infection (Karidis et al., 2015). However, additional *in vivo* experiments would be necessary to definitely draw this conclusion. These could include CCL_4_-stimulation approaches in a liver-specific A20 knockout background (Lee et al., 2000; Liu et al., 2013).

A couple of molecular mechanisms may explain the A20-mediated differential desensitization phenotype observed in our study. For example, A20 suppresses apoptotic JNK signaling in a TNF-dependent and independent manner via induction of ASK1 degradation (Won et al., 2010). Since A20 is efficiently induced by TNF administration it is tempting to speculate that the loss of ASK1 might shift the cellular response from apoptosis to survival and proliferation. Our data include an additional mechanistic level to this observation. ASK1 can phosphorylate JNK and the physical interaction between A20 and ASK1 has been demonstrated (Tobiume et al., 2001; Won et al., 2010). We here showed that TNF affects the interaction between A20 and ASK1, which could explain the ASK1-dependent phosphorylation of JNK at specific periods after TNF administration. The underlying molecular mechanism for this precise and dynamic fine-tune regulation is not understood, however, the natural oscillations observed in the activation dynamics of both JNK and NF-κB could be partly explained in terms of synchrony / asynchrony ASK1:A20 dynamics as observed in this study (Ashall et al., 2009).

Alternatively, A20 has been demonstrated to mediate RIPK1 ubiquitination and differential activation of the effector caspase-8. Here the ubiquitination activity of A20 protects from apoptosis after TRAIL treatment due to the reduced transfer of pro-caspase-8 to biologically active caspase-8 (Dong et al., 2012). However, it is not clear if these mechanisms are involved in the differential desensitization phenotype in hepatocellular cells after administration of low cytokine amounts.

In addition, our results indicate that the mode of TNF stimulation (single vs. repeated treatment) affects the cellular outcome. While single cytokine administration leads to temporary activation of p65 and JNK phosphorylation, the repeated stimulation with suboptimal (and physiological) TNF levels partly overcomes negative feedback regulation of JNK, while an effective inhibition of the NF-κB system is possible (Figure 4). These results reinforce the concept that different stimuli in space and time define cause different effects under physiological and/or pathophysiological conditions (Ashall et al., 2009). Our data suggest that A20 might represent one molecular switch how cells may discriminate between NF-κB and JNK signaling. This dynamic physical ASK1:A20 interaction might explain the oscillatory or sustained JNK activity and as a physiological consequence the switch between survival and apoptosis.

Together, our study suggests an A20-dependent mechanism, which may explain how hepatocytes differentially activate downstream effector pathways upon repeated stimulation with physiological TNF concentrations. Continuous availability of high TNF concentrations under pathophysiological conditions may uncouple this fine-tune regulation and therefore participate in the development of liver diseases. Future work might include the role of scaffold proteins (e.g., IQGAP2 and SQSTM1) and how their the subcellular localization might affect NF-κB and JNK pathway activity dependent on the cellular context (inflammation, loss of cell polarity). Knowledge on the spatio-/temporal regulation will help to define the specific responses of signaling pathways in healthy and diseased cells.

## Author contributions

FP and MB performed the experiments. TL, PS, FP, and KBr designed the experiments did data interpretation and wrote the paper. NH, UK, KBe, and SS performed computational modeling.

### Conflict of interest statement

The authors declare that the research was conducted in the absence of any commercial or financial relationships that could be construed as a potential conflict of interest.

## Supplementary material

Supplementary Figure S1 TNF-induced dynamics in Hep56 cells. **(A)** Western immunoblots of protein extract isolated from Hep56 cells after administration of TNF (10 ng/ml) for the indicated time-points. **(B)** Relative protein amounts of NF-κB and JNK pathways constituents in Hep56 cells after single TNF administration (10 ng/ml). **(C)** Relative mRNA levels of IκBα and A20 after single TNF stimulation. Bars in panel **(C,D)** represent standard errors.

Supplementary Figure S2 Comparison of NF-κB and JNK pathway activity in Hep56 cells after single and multiple TNF stimulation. This experiment was performed as described for Hepa1-6 cells (Figure 4). For this analysis, only time points were chosen, which showed differential signal amplitude between NF-κB and JNK in Hepa1-6 cells (untreated, 10, 70, and 130 min after TNF administration). **(A)** Single TNF treatment of Hep56 cells (1 × 1 ng/ml). **(B)** Triple TNF treatment Hep56 cells with 1 × 1 ng/ml followed by 2 × 2.5 ng/ml. **(C)** Triple TNF treatment Hep56 cells with 1 × 1 ng/ml followed by 2 × 5 ng/ml. For all graphs signals for p65/phospho-p65, and JNK/phospho-JNK were quantified and normalized to the respective loading control (actin). Bars represent standard errors.

Supplementary Figure S3 Confirmation of transfection efficiency of pCMV6 vectors. **(A)** Six technical replicates of Hepa1-6 clones transfected with pCMV6-A20 were tested for the overexpression of pMax-GFP. Test was repeated after establishment of individual clones (six clones are shown). **(B)** Brightfield and immunofluorescence pictures of Hepa1-6 cells after transient transfection of the pMax-GFP vector. Note that the majority of cells show moderate to strong positivity for GFP. **(C)** FACS analysis confirms that about 80% of all cells are positive for GFP.

Supplementary Figure S4 Changes in A20 expression do not affect p65 activity. **(A)** A20 overexpression does not affect p65 or its target gene IκBα. Transcript levels of A20, IκBα, and p65 were measured by real-time PCR. **(B)** Luciferase assay illustrates that A20 overexpression does not change the activity of p65. Bars represent standard errors.

Supplementary Figure S5 Analysis of HCC apoptosis after A20 inhibition and TNF stimulation. A20 was inhibited in Hep56 cells by gene-specific siRNA transfection. Twenty-four hours later cells were treated with TNF (10 ng/ml) and apoptosis was measured after indicated time points.

## Abbreviations

ASK1: Apoptosis Signal-regulating Kinase 1
HCC: Hepatocellular Carcinoma
IκBα: nuclear factor of Kappa Light polypeptide gene enhancer in B-cells inhibitor alpha
IL: Interleukin
MAP3K/MEKK7: Mitogen-Activated Protein Kinase Kinase Kinase 7
LSEC: Liver Sinusoidal Endothelial Cells
ODE: Ordinary Differential Equation
RIPK1: Receptor Interacting serine/threonine-Protein Kinase
ROS: Reactive Oxygene Species
TNF: Tumor-Necrosis Factor
TNFRI: TNF Receptor
TNFAIP3: TNF-Induced Protein 3.
