# Supplementary figures and images for: A20/TNFAIP3 Discriminates Tumor Necrosis Factor (TNF)-Induced NF-κB from JNK Pathway Activation in Hepatocytes
Source: Front Physiol. 2017 Aug 23;8:610. doi: 10.3389/fphys.2017.00610 (PMC5572400; doi:10.3389/fphys.2017.00610)

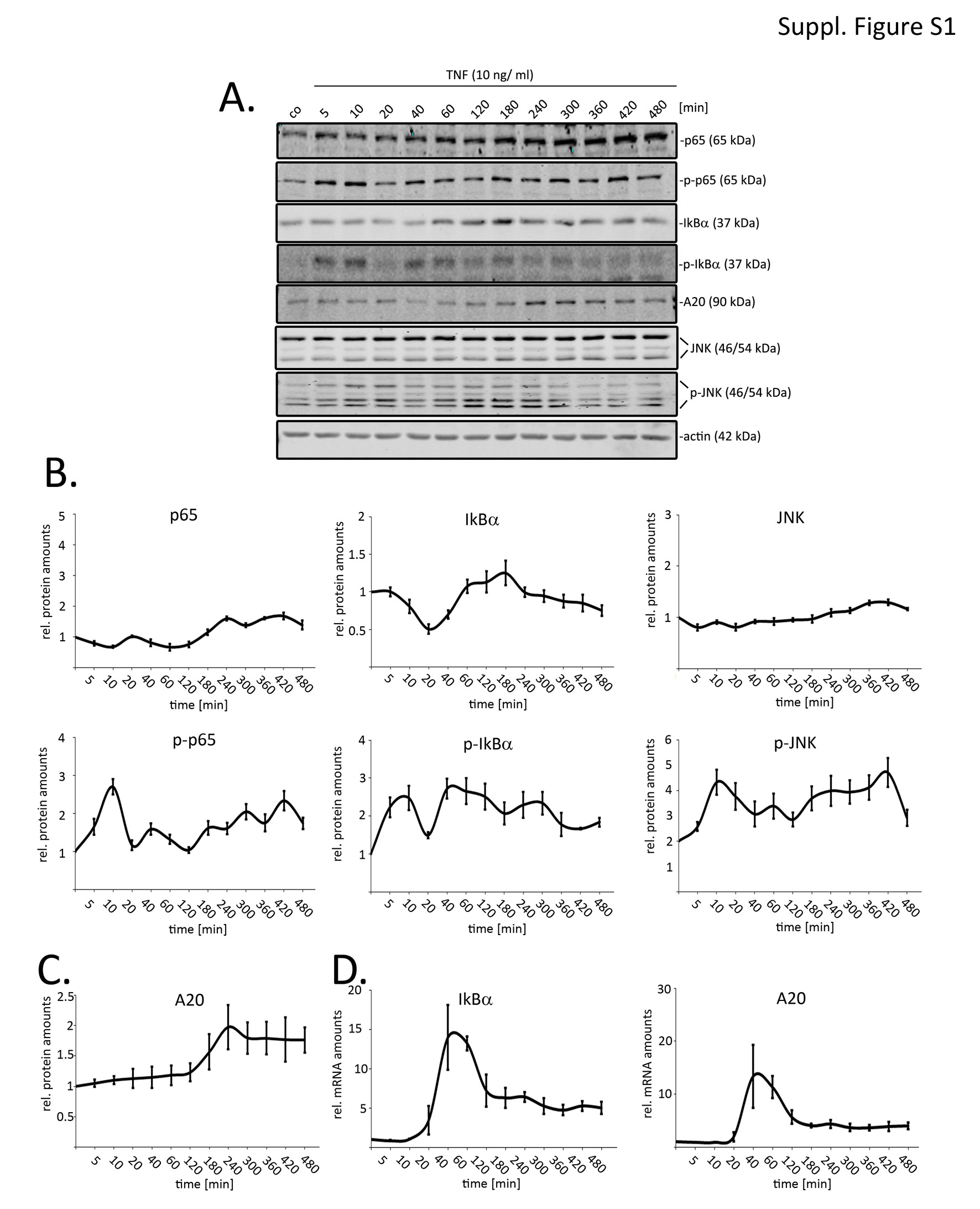

Supplement: Supplementary Figure S1 — TNF-induced dynamics in Hep56 cells. (A) Western immunoblots of protein extract isolated from Hep56 cells after administration of TNF (10 ng/ml) for the indicated time-points. (B) Relative protein amounts of NF-κB and JNK pathways constituents in Hep56 cells after single TNF administration (10 ng/ml). (C) Relative mRNA levels of IκBα and A20 after single TNF stimulation. Bars in panel (C,D) represent standard errors. [file Image1.JPEG]

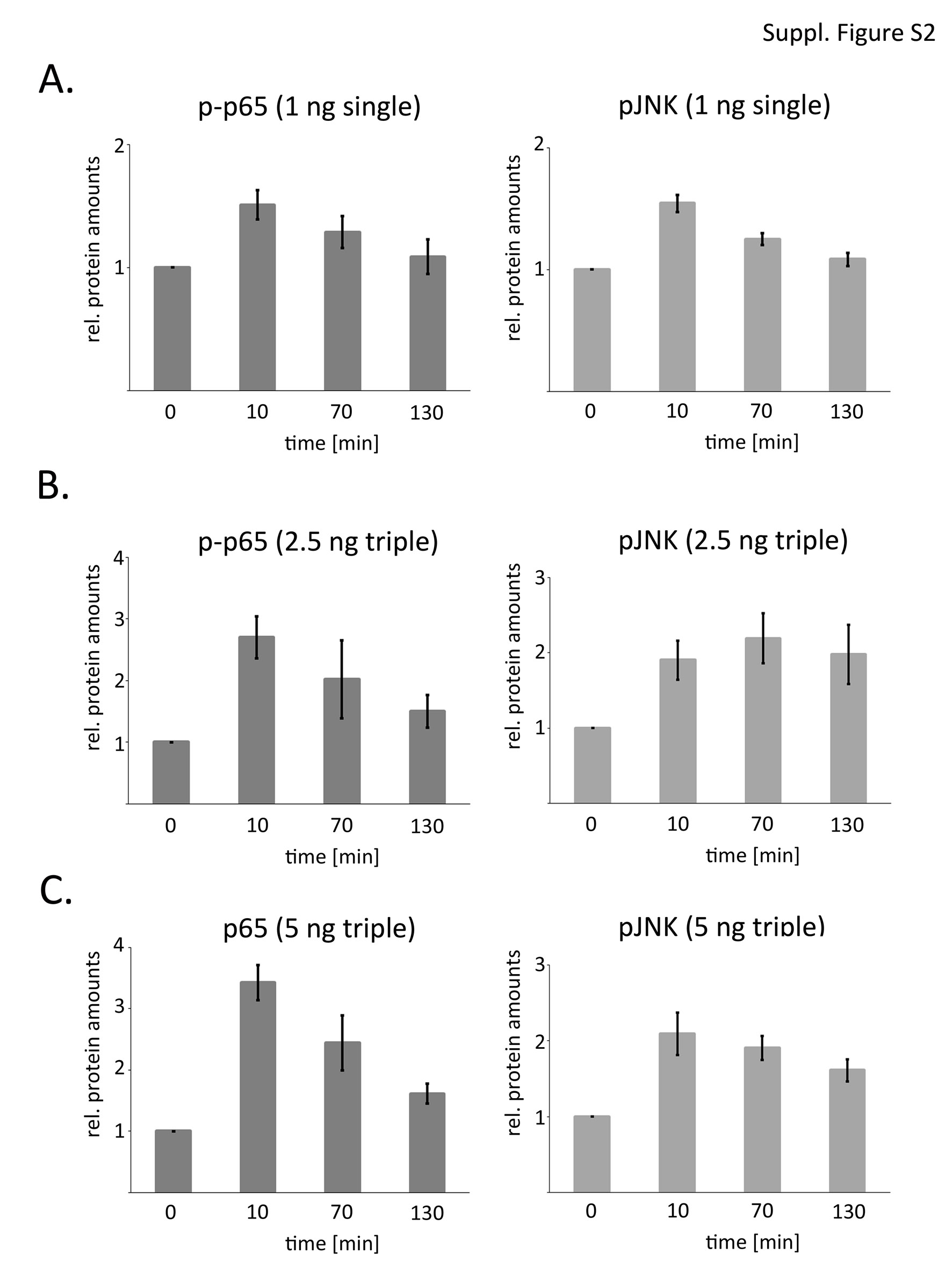

Supplement: Supplementary Figure S2 — Comparison of NF-κB and JNK pathway activity in Hep56 cells after single and multiple TNF stimulation. This experiment was performed as described for Hepa1-6 cells (Figure 4). For this analysis, only time points were chosen, which showed differential signal amplitude between NF-κB and JNK in Hepa1-6 cells (untreated, 10, 70, and 130 min after TNF administration). (A) Single TNF treatment of Hep56 cells (1 × 1 ng/ml). (B) Triple TNF treatment Hep56 cells with 1 × 1 ng/ml followed by 2 × 2.5 ng/ml. (C) Triple TNF treatment Hep56 cells with 1 × 1 ng/ml followed by 2 × 5 ng/ml. For all graphs signals for p65/phospho-p65, and JNK/phospho-JNK were quantified and normalized to the respective loading control (actin). Bars represent standard errors. [file Image2.JPEG]

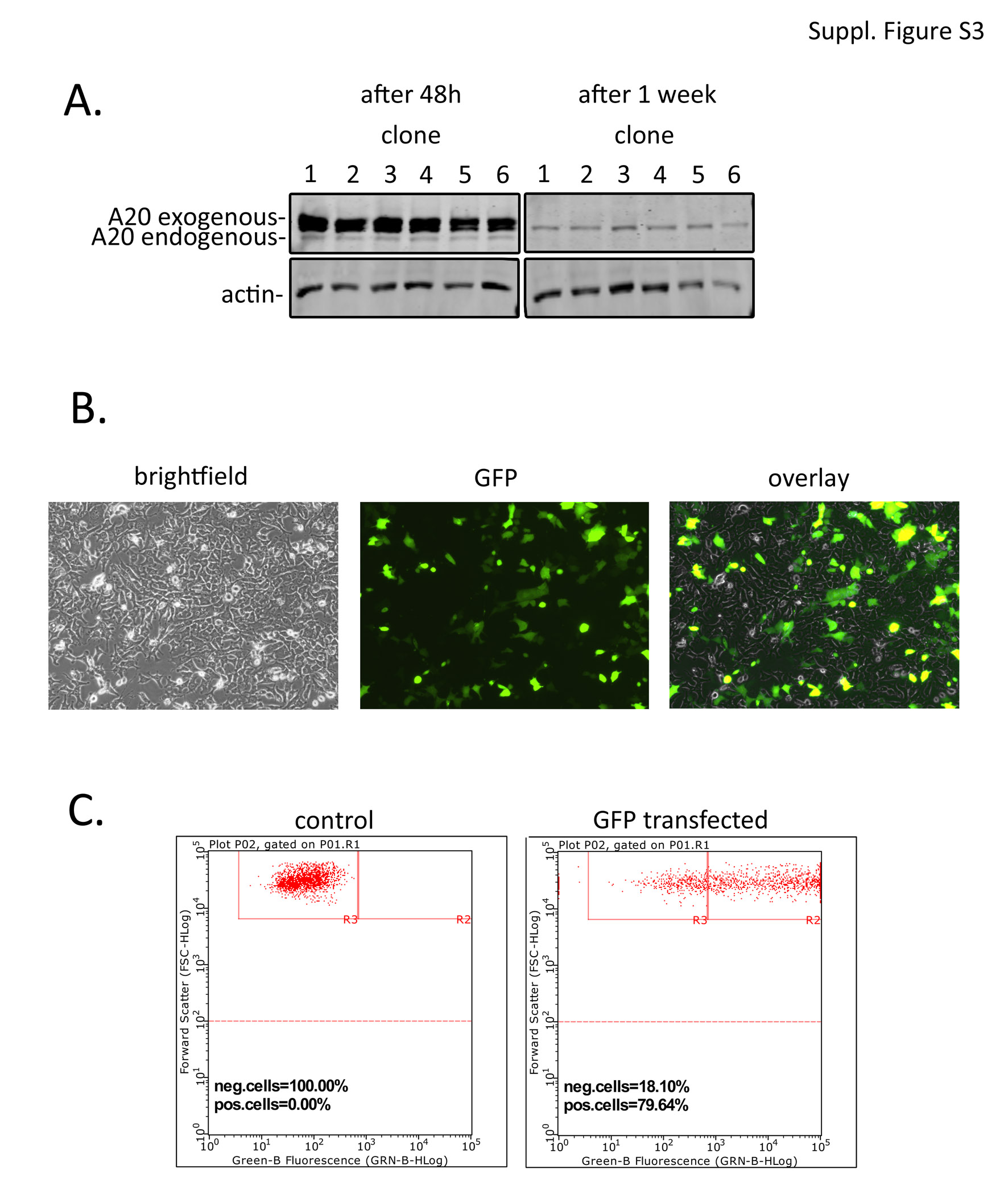

Supplement: Supplementary Figure S3 — Confirmation of transfection efficiency of pCMV6 vectors. (A) Six technical replicates of Hepa1-6 clones transfected with pCMV6-A20 were tested for the overexpression of pMax-GFP. Test was repeated after establishment of individual clones (six clones are shown). (B) Brightfield and immunofluorescence pictures of Hepa1-6 cells after transient transfection of the pMax-GFP vector. Note that the majority of cells show moderate to strong positivity for GFP. (C) FACS analysis confirms that about 80% of all cells are positive for GFP. [file Image3.JPEG]

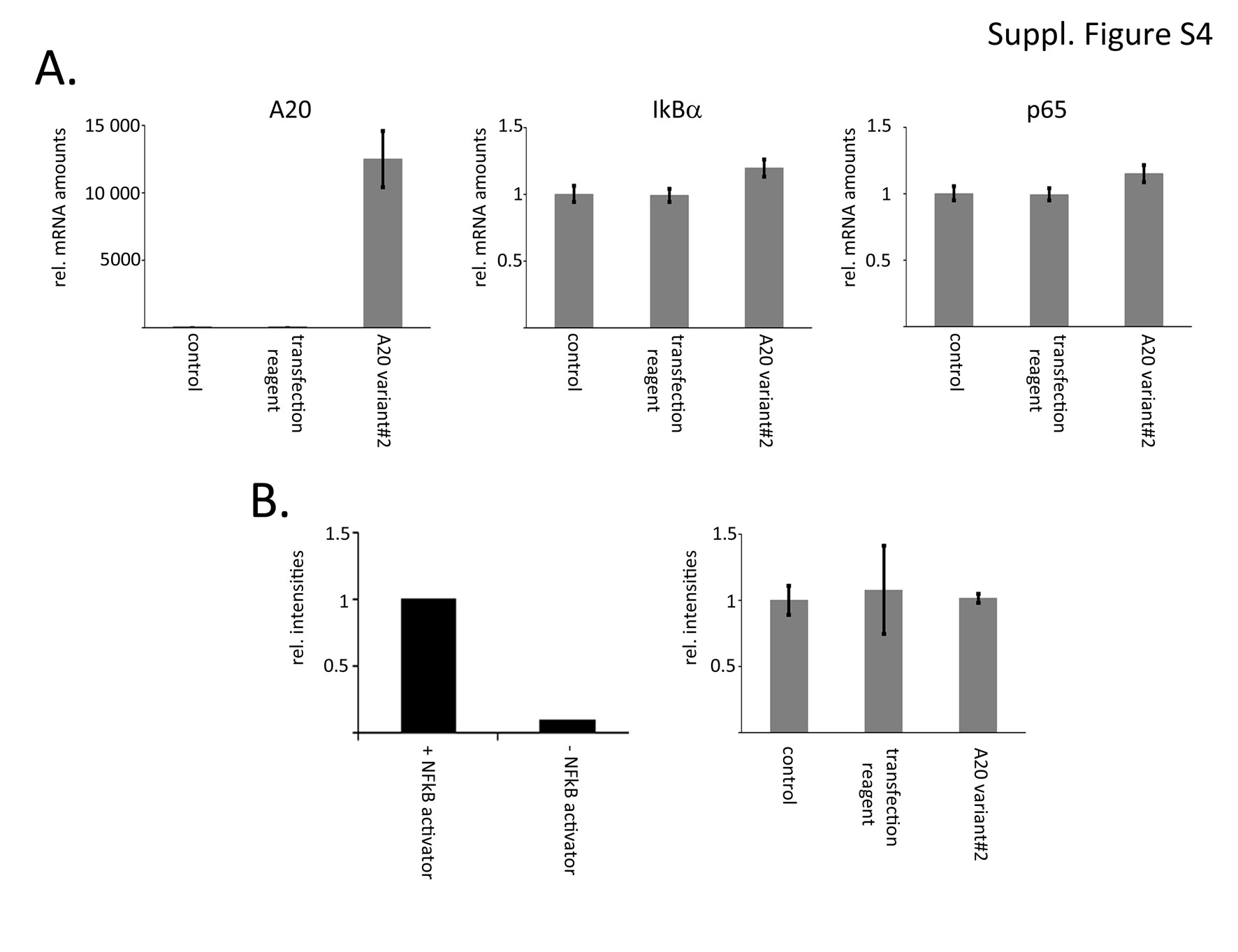

Supplement: Supplementary Figure S4 — Changes in A20 expression do not affect p65 activity. (A) A20 overexpression does not affect p65 or its target gene IκBα. Transcript levels of A20, IκBα, and p65 were measured by real-time PCR. (B) Luciferase assay illustrates that A20 overexpression does not change the activity of p65. Bars represent standard errors. [file Image4.JPEG]

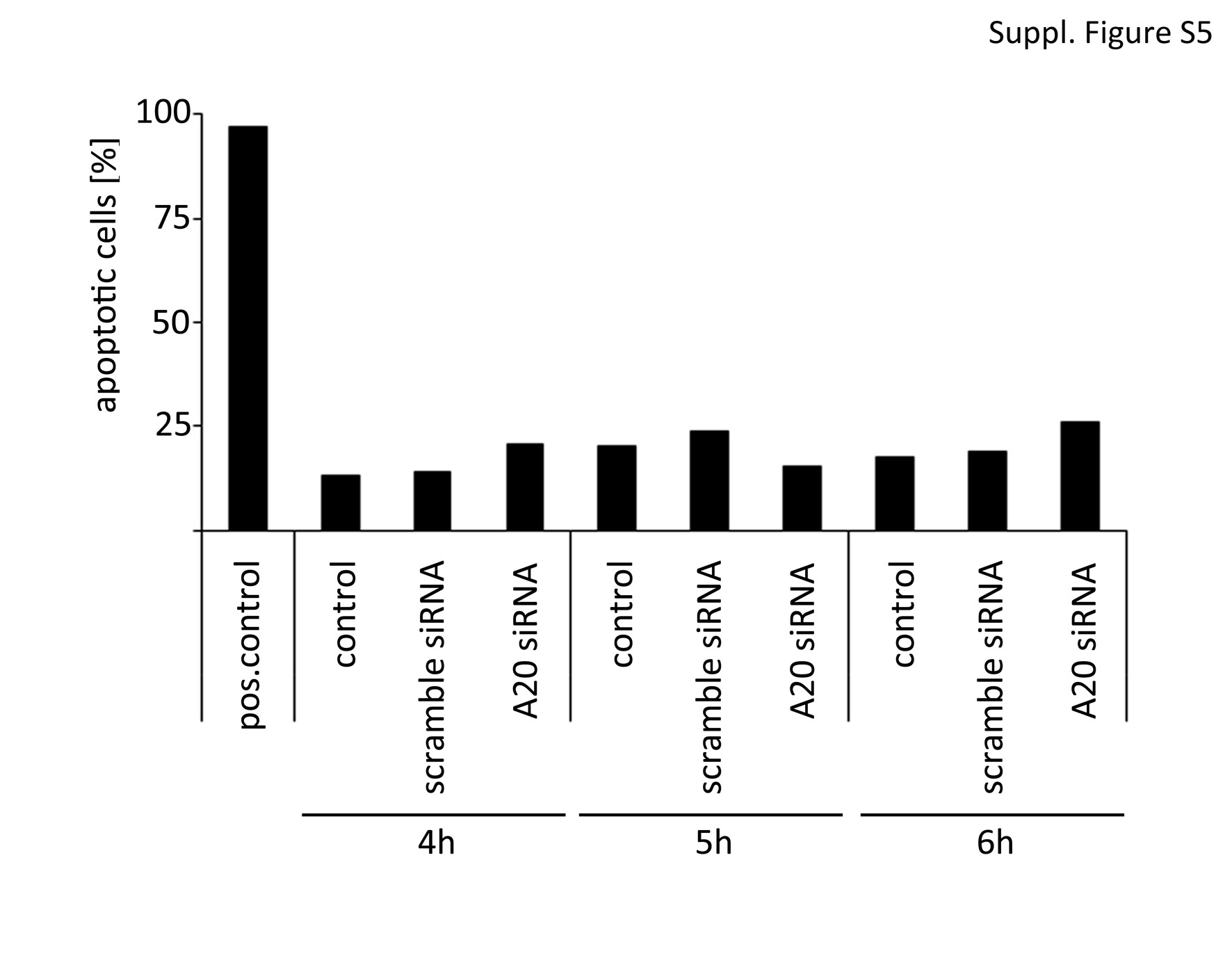

Supplement: Supplementary Figure S5 — Analysis of HCC apoptosis after A20 inhibition and TNF stimulation. A20 was inhibited in Hep56 cells by gene-specific siRNA transfection. Twenty-four hours later cells were treated with TNF (10 ng/ml) and apoptosis was measured after indicated time points. [file Image5.JPEG]
